# Infusion of freshly isolated autologous bone marrow derived mononuclear cells prevents endotoxin-induced lung injury in an *ex-vivo *perfused swine model

**DOI:** 10.1186/scrt174

**Published:** 2013-03-04

**Authors:** Mauricio Rojas, Richard E Parker, Natalie Thorn, Claudia Corredor, Smita S Iyer, Marta Bueno, Lyle Mroz, Nayra Cardenes, Ana L Mora, Arlene A Stecenko, Kenneth L Brigham

**Affiliations:** 1 Dorothy P. and Richard P. Simmons Center for Interstitial Lung Diseases, University of Pittsburgh School of Medicine, 3459 Fifth Avenue, Pittsburgh, PA 15213, USA; 2 Division of Pulmonary, Allergy and Critical Care Medicine, University of Pittsburgh School of Medicine, 3459 Fifth Avenue, Pittsburgh, PA 15213, USA; 3 McGowan Institute for Regenerative Medicine, University of Pittsburgh School of Medicine, 3459 Fifth Avenue, Pittsburgh, PA 15213, USA; 4 Division of Pulmonary, Allergy and Critical Care Medicine, Emory University School of Medicine. 615 Michael Street, Atlanta, GA 30322, USA; 5 Center for Translational Research in the Lung, Emory University School of Medicine. 615 Michael Street, Atlanta, GA 30322, USA; 6 McKelvey Center for Lung Transplantation, Department of Medicine, Emory University School of Medicine, 615 Michael Street, Atlanta, GA 30322, USA; 7 Division of Pediatric Pulmonary, Allergy, Cystic Fibrosis and Sleep Medicine, Department of Pediatrics. Emory University School of Medicine, 2015 Uppergate Drive, Atlanta, GA 30322, USA; 8 Emory Georgia Tech Predictive Health Institute, Emory University School of Medicine, 615 Michael Street, Atlanta, GA 30322, USA

## Abstract

**Introduction:**

The acute respiratory distress syndrome (ARDS), affects up to 150,000 patients per year in the United States. We and other groups have demonstrated that bone marrow derived mesenchymal stromal stem cells prevent ARDS induced by systemic and local administration of endotoxin (lipopolysaccharide (LPS)) in mice.

**Methods:**

A study was undertaken to determine the effects of the diverse populations of bone marrow derived cells on the pathophysiology of ARDS, using a unique *ex-vivo *swine preparation, in which only the ventilated lung and the liver are perfused with autologous blood. Six experimental groups were designated as: 1) endotoxin alone, 2) endotoxin + total fresh whole bone marrow nuclear cells (BMC), 3) endotoxin + non-hematopoietic bone marrow cells (CD45 neg), 4) endotoxin + hematopoietic bone marrow cells (CD45 positive), 5) endotoxin + buffy coat and 6) endotoxin + *in vitro *expanded swine CD45 negative adherent allogeneic bone marrow cells (cultured CD45neg). We measured at different levels the biological consequences of the infusion of the different subsets of cells. The measured parameters were: pulmonary vascular resistance (PVR), gas exchange (PO_2_), lung edema (lung wet/dry weight), gene expression and serum concentrations of the pro-inflammatory cytokines IL-1β, TNF-α and IL-6.

**Results:**

Infusion of freshly purified autologous total BMCs, as well as non-hematopoietic CD45(-) bone marrow cells significantly reduced endotoxin-induced pulmonary hypertension and hypoxemia and reduced the lung edema. Also, in the groups that received BMCs and cultured CD45neg we observed a decrease in the levels of IL-1β and TNF-α in plasma. Infusion of hematopoietic CD45(+) bone marrow cells or peripheral blood buffy coat cells did not protect against LPS-induced lung injury.

**Conclusions:**

We conclude that infusion of freshly isolated autologous whole bone marrow cells and the subset of non-hematopoietic cells can suppress the acute humoral and physiologic responses induced by endotoxemia by modulating the inflammatory response, mechanisms that do not involve engraftment or trans-differentiation of the cells. These observations may have important implications for the design of future cell therapies for ARDS.

## Introduction

Respiratory diseases kill more than 400,000 Americans each year and significantly reduce the quality of life for millions more. The National Heart, Lung and Blood Institute (NHLBI) estimated that in 2009 the annual cost of providing healthcare related to all respiratory conditions, excluding lung cancer, was $113 billion [1]. Acute Respiratory Distress Syndrome (ARDS) is a very common clinical entity and a major cause of morbidity and mortality in the critical care setting. Historically, ARDS has been associated with mortality ranging from 40% to 60%, with worse outcomes in the older population. In the US alone, 150,000 new cases of ARDS occur every year [2]. Moreover, ARDS has a significant impact on long-term disability and adverse psycho-social outcomes in survivors [3]. According to the Berlin definition of ARDS, the diagnostic criteria for ARDS rely on four categories: (i) timing: within one week of a known clinical insult or new or worsening respiratory symptoms; (ii) radiographic: bilateral opacities not fully explained by effusions, lobar/lung collapse, or nodule; (iii) origin of lung edema: respiratory failure not fully explained by cardiac failure or fluid overload; and (iv) oxygenation impairment. Consequently, ARDS was divided into three categories according to the degree of hypoxemia: mild, moderate and severe. Thus, the Berlin definition eliminated the concept of acute lung injury (ALI), which now falls into the category of mild ARDS. ARDS always results from another severe underlying disease. The range of diseases causing ARDS is broad, and they may also damage organs other than the lungs, but the lung injury invariably dominates the clinical picture. Sepsis is the most common condition leading to ARDS. In mammals, ARDS is initiated by an acute inflammatory response to a physical trauma or infection [4-9] followed by sequestration of neutrophils in the lung, lung edema, and up-regulation of inflammatory mediators both locally and systemically.

Bone marrow derived stem cells can be divided in two groups: hematopoietic stem cells (HSC) and mesenchymal stromal stem cells (MSC). Bone marrow derived MSC were first described in the early 1970s by Friedenstein and collaborators [10-13]. They are defined as clonal, plastic adherent cells capable of differentiating into cells of mesenchymal origin. These cells are also able to support hematopoiesis in culture providing extracellular matrix, cytokines, and growth factors to the HSC. The ultimate characterization of mesenchymal stem cells has been a complicated issue since there are no specific cell surface markers. Enrichment of mesenchymal stem cells from crude bone marrow suspensions is achieved by selecting a plastic-adherent population that expresses neither hematopoietic nor endothelial cell surface markers but is positive for the expression of adhesion and stromal markers [14]. The criterion for establishing MSC phenotype is to use adherent cells that: (i) express CD44, CD73, CD90 and CD105; (ii) lack the expression of hematopoietic markers, such as CD45, CD34 and CD31; and (iii) confirm their plasticity by the ability of the cells to differentiate into adipocytes, osteocytes, and chondrocytes in a multilineage differentiation assay [15]. Cells used in the present work are non-hematopoietic bone marrow adherent cells. However, because the cells were not completely characterized, mainly because of the lack of specific antibodies to all the markers required, we limit the denomination to *in vitro *expanded swine CD45 negative adherent allogeneic bone marrow cells (cultured CD45neg)

Cell-based therapy in experimental models of ARDS and sepsis has been the focus of intense investigation since the publication of our original work in 2007 [16]. In our initial observation, we defined the beneficial effects of the systemic administration of mesenchymal stem cells to control ARDS mainly by their anti-inflammatory properties. Multiple groups have reported similar observations where the protective effect has been demonstrated in different animal models and human *ex-vivo *lungs [17-22]. The protective effects of mesenchymal stem cells are attributed to several mechanisms including secretion of the anti-inflammatory cytokines IL-10 and TGF-β, and Keratinocyte Growth Factor (KGF) and elaboration of antibacterial peptides. As a result of these most recent studies, mesenchymal stem cells are increasingly recognized for having complex interactions with the host immune system and share properties with cells of the innate immune system. Given the pleiotropic effects of these stem cells in the setting of ARDS and sepsis, there has been considerable interest in initiating translational studies in this field.

In the present pre-clinical model, we found that infusion of freshly purified autologous nuclear bone marrow cell preparations or non-hematopoietic CD45 (-) bone marrow cells has similar protective characteristics to *in vitro *expanded CD45neg by moderating the very early pathophysiological events in endotoxin-induced lung injury. They also were able to moderate pulmonary hypertension and hypoxemia and, in the case of bone marrow cells and cultured CD45neg, attenuated the increase in serum levels of the pro-inflammatory cytokines TNF-α and IL-1β.Neither autologous buffy coat cells nor hematopoietic CD45 (+) cells had those effects. We conclude that similar to cultured CD45neg, freshly isolated nucleated bone marrow cells and the subpopulation of non-hematopoietic bone marrow cells can interact with the immune system to modulate inflammation and control lung injury resulting from severe endotoxemia.

## Methods

### The *in situ *piglet preparation

We have previously described an *ex-vivo *perfusing piglet preparation, which permits perfusion restricted to the lung and the liver using autologous blood [23]. Piglets were anesthetized, a tracheostomy was performed, and cannulas were placed in a carotid artery and a jugular vein. After heparinization, the animals were ex-sanguinated and the blood was used to fill the blood reservoir and the perfusion circuit. The lungs were perfused at a constant flow rate of 40 ml/kg/minute (up to 400 ml/min); half of the blood in the efferent circuit transverses the liver before perfusing the lung. In all the preparations, lungs were ventilated under the same conditions with a piston ventilator (Harvard Apparatus, Dover, MA, USA) with room air and 5% CO_2_. The minute ventilation was adjusted to maintain perfusate pH between 7.35 and 7.45. Throughout the experiments, pressures in the pulmonary artery, left atrium and portal vein and flow in and out of both organs was monitored continuously and the data were stored in a computer for later analysis.

The study adhered to National Institute of Health guidelines on the use of experimental animals and was approved by the institutional animal care and use committee of Emory University. Animals were maintained in the Emory University Division of Animal Resources, an AAALAC approved facility.

### Experimental protocols and data collection

Six groups of experiments were performed with four to six animals in each group. The groups were designated as: 1) endotoxin alone, 2) endotoxin + freshly isolated autologous bone marrow nuclear cells, 3) endotoxin + non-hematopoietic bone marrow autologous cells CD45(-), 4) endotoxin + hematopoietic bone marrow cells CD45(+), 5) endotoxin + buffy coat and 6) endotoxin + *in vitro *expanded swine CD45 negative adherent allogeneic bone marrow cells (cultured CD45neg). For all the groups, 5 μg/kg body weight of endotoxin was used (Serotype O55:B5 Difco, Detroit, MI, USA), directly into the blood reservoir fifteen minutes after endotoxin cells were added to the blood reservoir. A total of 100 x10^6 ^whole bone marrow cells were used. In the case of the subpopulations, CD45 (-), CD45(+), buffy coat and cultured CD45neg, 50 x10^6 ^purified cells were perfused in each preparation. Blood samples were collected before and after endotoxin administration. Blood gas analysis was performed every 15 minutes for the duration of the experiment using a portable blood gas analyzer (Rapidlab 248, Diamond Diagnostics, East Walpole, MA, USA). Serum samples were separated and stored at -80^o ^for measurement of TNF-α, IL-6 and IL-1β. Automated cell count and differential counts were performed every 15 minutes using a device which is specifically designed for use in laboratory animals (Hemevet, CDC Technologies, Oxford, CT, USA). At each time point, we recorded pressures and flows in and out of the lungs and flow to the liver. Pulmonary vascular resistance (PVR) was calculated as in-flow pressure (pulmonary artery pressure) minus out-flow pressures (left atrial pressure) divided by total blood flow through the lungs and expressed in cm H_2_O/ml/min. Temperature was measured continuously and kept between 38°C and 40°C by adjusting the temperature of the water in the water-jacket surrounding the reservoir.

Lung and liver tissue specimens were obtained at three time points (30 minutes before endotoxin and 45 minutes and 135 minutes after endotoxin) for the determination of histology, cell engraftment and gene expression in a microarray system. The study was terminated two and a half hours after endotoxin administration and both lungs were taken for determination of wet-to-dry weight ratio.

### Generation and administration of cells

Briefly, fresh swine bone marrow derived cells were isolated by mechanically extracting bone marrow from both femurs of the animal while the preparation was being set up. The harvested bone marrow was cut in small fragments and cells detached by continuous vortex for 30 seconds followed by 1X trypsin-ethylenediaminetetraacetic acid (EDTA) for 2 minutes. Cell suspensions were centrifuged for 2 minutes at 700 RPM to separate insoluble material. Supernatant was collected and cells transferred to a 50 ml conical tube where cells were washed twice with PBS containing penicillin-streptomycin. Red blood cells were lysed by re-suspending the cell pellet with 3 ml of sterile water. After 15 seconds lysis was stopped by adding 27 ml of 10X PBS for a final concentration of 1X PBS. Cells were washed twice with sterile PBS and counted in a hemocytometer. To isolate CD45 (+) and CD45 (-) cells, 300 × 10^6 ^bone marrow cells were transferred into a 15 ml conical tube and incubated with 35 μg of fluorescein isothiocyanate (FITC) conjugated mouse anti-porcine CD45 (ABD Serotec, Oxford, UK) for 10 minutes at 4#176;C. Cells were washed twice with cold MACS buffer (PBS +0.5% BSA+2mM EDTA). After washing, cells were re-suspended in 1.5 ml MACS buffer and incubated with 300 μl of anti-FITC microbeads (Myltenyi Biotec, Auburn, CA, USA) for 15 minutes at 4#176;C. CD45 (-) cells were sorted using LD columns (Myltenyi Biotec, Auburn, CA, USA). Cell sorting was repeated once to ensure the purity of the cells. Cells that were retained in the column were used as CD45 (+) cells. After the final elution, cells were washed twice with sterile saline solution. Before infusion, cells were counted in a hemocytometer with Trypan blue to determine cell numbers and viability.

Autologous buffy coat cells were obtained by separating leukocytes from fresh heparinized peripheral blood by osmotic gradient using HISTOPAQUE^® ^1083 (Sigma, St Louis, MO, USA). Twenty ml of fresh blood was centrifuged and red blood cells lysed. Cells were re-suspended in 20 ml of PBS and placed on top of 20 ml of Histopaque 1083. The opaque interface, containing the mononuclear cell band, was collected using a Pasteur pipet and transferred into a 50 ml conical centrifuge tube. Cells were washed twice with PBS. After the last wash, cells were counted to determine viability and cell numbers.

Swine CD45 negative adherent allogeneic bone marrow cells (cultured CD45neg) were isolated by negative selection of CD45(+) cells, using an anti-CD45-FITC antibody, followed by anti-FITC-conjugated magnetic beads (Miltenyi Biotec). Cultured CD45neg cells were expanded *in vitro *in 175 cm^2 ^flasks in Iscove's modified Dulbeccos's medium (IMDM) containing 9% fetal bovine serum (FBS, Atlanta, Biologicals, Norcross, GA, USA), 9% horse serum, and 1% penicillin-streptomycin. Cells were harvested at 70% confluence using 0.25% trypsin. For *in vivo *infusion, cells were detached using 0.25% trypsin at 37°C for 5 minutes. Trypsin was neutralized by adding IMDM with 10% serum. The cell suspension was centrifuged and suspended in sterile PBS (without Ca and Mg). After two additional washes with PBS, cells were counted in a hemocytometer with trypan blue and were re-suspended in PBS at 5 × 10^6 ^cells per ml final concentration.

To track the cells in the swine preparation, BMCs were surface-labeled using PKH (Sigma. St Louis MO, USA). Prior to infusion, 10 × 10^7 ^cells were re-suspended in 5 ml of staining buffer and 50 μl of PKH solution were added. After incubating for 5 minutes at room temperature, the reaction was stopped by adding 10 ml of complete FBS. Cells were washed twice with 50 ml of sterile saline solution. Before infusion, cells were counted to determine viability and cell numbers.

### FACS analysis

Blood samples obtained from preparations were collected at different time points. Red blood cells were lysed by hypotonic shock and samples analyzed for PKH-26 staining on a fluorescence-assisted cell sorting (FACS) Excalibur (Becton Dickinson, San Jose, CA, USA), using CellQuest software (Becton Dickinson, San Jose, CA, USA) and analyzed using FlowJo software (Tree Star, San Carlos, CA, USA).

### Immunohistochemistry

Pig lungs were placed in 2% paraformaldehyde and processed for paraffin embedding. Sections (5 mm) were cut, mounted on the slides, and subjected to antigen retrieval in a decloaking chamber (BioCare Medical, Gaithersburg, MD, USA). Endogenous peroxidase activity was quenched with 3% peroxide for 5 minutes. Slides were incubated with anti-myeloperoxidase antibody (MPO) (Abcam Cambridge, MA, USA) overnight at 4#176;C. Immune complexes were visualized with biotinylated secondary antibody and 3,3'-diaminobenzidine tetrahydrochloride using the streptavidin-biotin complex method.

### Cytokine measurements

Plasma concentrations of TNF-α, IL-6 and IL-1β were measured separately using Quantikine^® ^ELISA kits (R&D Systems, Minneapolis, MN, USA) according to the manufacturer's instructions. Briefly, samples were dispensed into 96-well micro-titer plates that are precoated with porcine monoclonal or polyclonal antibodies specific to the above cytokines. After washing away any unbound substances, an enzyme (horseradish peroxidase)-linked monoclonal (TNF-α) or polyclonal (IL-6 and IL-1β) antibody specific to the above cytokines was added to the wells. Following a wash to remove any unbound antibody-enzyme reagent, a substrate solution (hydrogen peroxide/tetramethylbenzidine) was added to the wells. The reaction was terminated with a stop buffer and absorbance read at 450 nm using an MRX Revelation (Dynex Technologies, Chantilly, VA, USA) multi-well plate reader. Values of IL-6, TNF-α and IL-1β were determined by reference to a standard curve constructed using the porcine proteins and computer software capable of generating a four parameter logistic curve-fit.

### Measurements of lung water

At the completion of each experiment, both lungs were harvested and homogenized. The homogenate was weighed (wet weight), then dried to constant weight in a microwave oven and reweighed (dry weight). The ratio of wet/dry lung weight was calculated as a measure of the amount of lung water (edema) that was present.

### Liver RNA isolation and Affymetrix GeneChip array

We used a swine specific microarray chip from Affymetrix with 27,000 genes represented. RNA was isolated from liver tissue samples taken 30 minutes after endotoxin in a liver-lung perfused preparation or after 30 minutes of perfusion without addition of endotoxin as a control. We performed microarray analyses of these samples and calculated the ratio of expression in the sample from the endotoxin-treated preparation to the control sample. Liver tissue samples from both endotoxin and endotoxin-bone marrow cell groups were homogenized in TRIzol reagent (Gibco BRL, Gaithersburg, MD, USA), and RNA was extracted according to the instructions of the manufacturer. RNA precipitates were briefly air-dried then subjected to an RNeasy protect mini kit and DNAse I treatment (Qiagen, Miami, FL, USA). Total RNA concentration was measured spectrophotometrically at 260 and 280 nm and dissolved in RNAse-free water to 1 µg/µL and stored at -80°C. For control of concentration and integrity, 1 µg of each RNA sample was subjected to formaldehyde-agarose electrophoresis.

Total RNA (5 µg) was used for microarray analysis on swine Affymetrix chips. Preparation of complementary RNA, hybridization, scanning and image analysis of the microarrays were each performed according to the manufacturer's protocol (Affymetrix, Santa Clara, CA, USA) at the Emory Bio-Core facility with a final ArrayExpress accession: E-MTAB-1436

### Statistical analysis

Statistically significant differences were determined by using analysis of variance (ANOVA) and Tukey-Kramer for post hoc test. *P *<0.05 was considered significant. In most cases the physiological data are presented as change from baseline, with the baseline calculated as the mean of values at thirty minutes prior and immediately prior to administering endotoxin.

## Results

### Infusion of autologous bone marrow cells prevented lung inflammation induced by systemic administration of endotoxin

**Figure 1:**
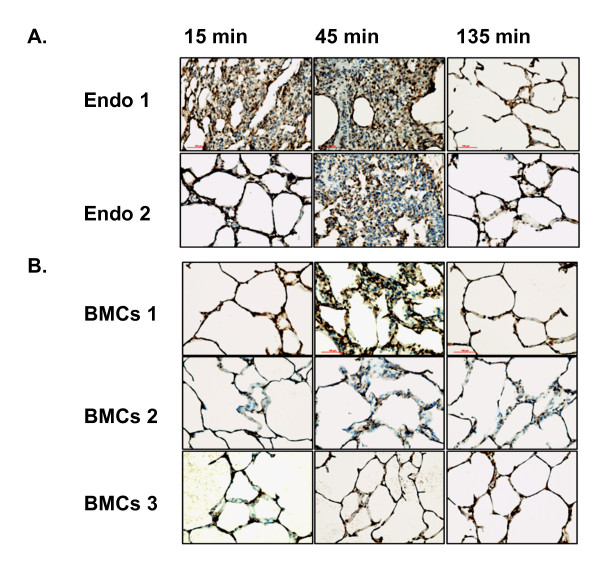
**Systemic administration of freshly isolated autologous whole bone marrow nuclear cells (BMC) modifies endotoxin (LPS) induced lung inflammation**. **A**. Lung biopsies were obtained at the indicated time points after endotoxin and stained with an anti-myeloperoxidase (MPO) antibody to detect neutrophils. Samples from two independent animals treated with endotoxin (Endo1 and 2) are shown. Note the significant alteration of lung architecture with increased inflammatory infiltrates and edema at 15 and 45 minutes after endotoxin. **B**. Histological lung sections stained with MPO from swine preparations infused with total BMCs 15 minutes after endotoxin. Three independent animals are shown (BMC 1, 2 and 3). Tissue samples were collected at the indicated time points after endotoxin. Notice reduction in the severity of inflammation and edema compared to endotoxin alone treated swine. LPS, lipopolysaccharide.

As one measure of the inflammatory response, we analyzed the influx of neutrophils into lungs by MPO immunostaining of histologic sections. As shown in Figure 1, lungs from animals receiving endotoxin had extensive infiltration of MPO-positive cells, most prominently at 45 minutes after administering endotoxin [23]. The marked alteration in lung architecture was largely resolved by 135 min after endotoxin. To investigate whether infusion of freshly isolated total BMCs into the lung reduced the lung inflammatory response, we added 100 × 10^6 ^cells of fresh isolated autologous BMCs to the reservoir 15 minutes after endotoxin. Histopathological analyses and MPO staining of lungs treated with BMCs showed reduced inflammatory infiltrates and tissue edema at the 45-minute time point (Figure 1) compared to animals receiving endotoxin alone.

### Trafficking of infused bone marrow cells in the lung after endotoxin-induced lung injury

**Figure 2:**
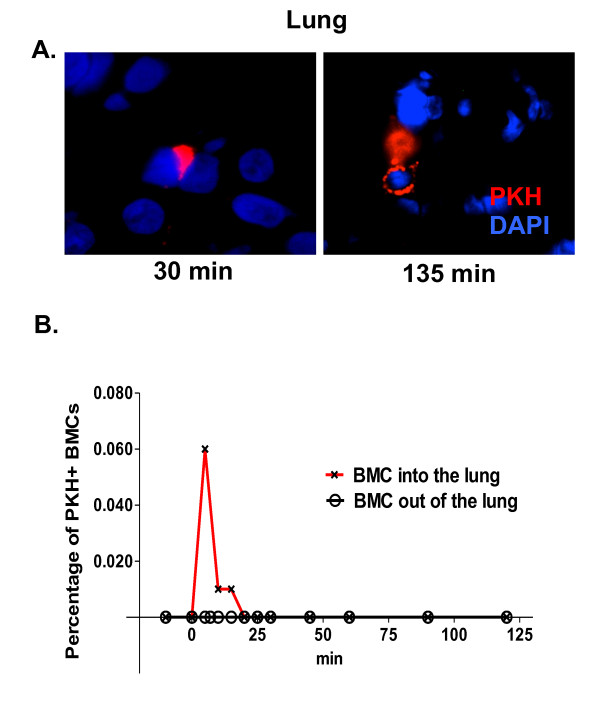
**Infused autologous bone marrow nuclear cells are retained in the lungs after endotoxin-induced acute lung injury**. To differentiate infused from endogenous cells, isolated cells were surface stained with PKH. To detected engrafted cells in the lung, a sample of lung tissue was collected at the end of the experiment and frozen in Tissue-Tek solution. **A**. Persistent localization of PKH staining bone marrow cells (red) in the lung following endotoxemia in the swine liver-lung perfused preparation (blue = nuclei). **B**. To demonstrate trapping of the cells in the lung, 0.5 cc blood sample was collected every 5 minutes from the pulmonary vein and artery, and analyzed by FACS. On B, there is a quantification of the percentage of PKH+ cells collected from blood before and after the lung. FACS, fluorescence-assisted cell sorting.

To determine the homing of whole bone marrow cells to the lung in response to endotoxin, BMCs were labeled with a fluorescent membrane dye, PKH26. Fifteen minutes after adding endotoxin to the reservoir, 100 × 10^6 ^labeled cells were added to the reservoir and the cell traffic was followed by collecting 0.5 ml blood samples from the pulmonary vein and pulmonary artery every 5 minutes until the end of the experiment. We detected few PKH labeled cells in histological sections from lungs (Figure 2A); however, a substantial number of labeled cells were detected by FACS analysis in the peripheral blood. Figure 2B shows higher numbers of labeled cells in afferent than in efferent circulation of the lungs during the early time points which is consistent with the retention of the cells in the lung after injury that has been observed in other animal models.

### BMCs infusion protects against endotoxin-induced pulmonary hypertension and hypoxemia

**Figure 3:**
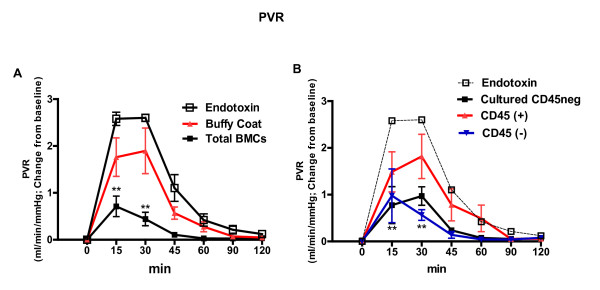
**Freshly isolated bone marrow nuclear cells moderated the increase in pulmonary vascular resistance induced by endotoxin**. **A**. Comparison of effects of the fresh bone marrow cells and the blood buffy coat cells on the pulmonary vascular resistance response. **B**. Endotoxin-induced PVR rapidly recovered in preparations in which CD45(-) and cultured CD45neg cells were included but persisted in the presence of CD45(+). (*n *= 6 in each group, **P <0.01). PVR, pulmonary vascular resistance.

Figure 3A shows attenuation of endotoxin-induced pulmonary hypertension by the infusion of freshly isolated autologous BMCs. Similarly, statistically significant diminution of the pulmonary vascular resistance response to endotoxin was observed when the subpopulations of non-hematopoietic CD45(-) bone marrow cells or swine CD45 negative adherent allogeneic bone marrow cells (cultured CD45neg) were administered. Neither buffy coat nor hematopoietic CD45(+) cells affected the endotoxin-induced pulmonary hypertension significantly (Figure 3B).

**Figure 4:**
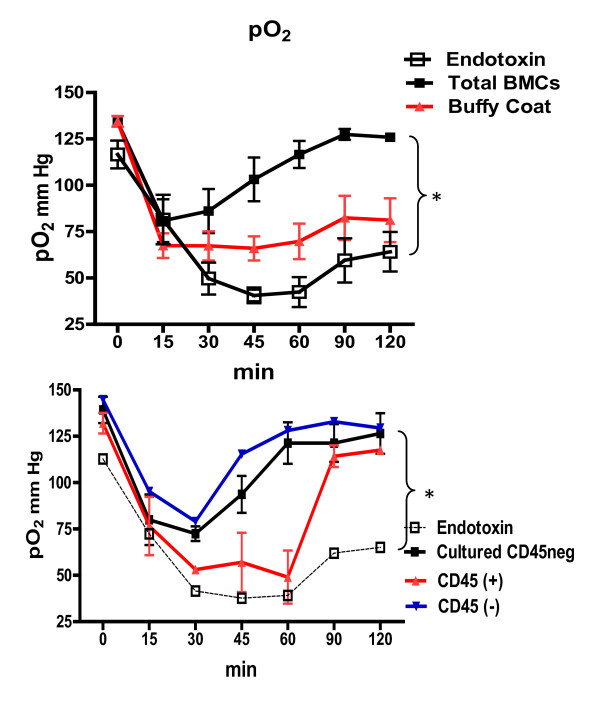
**Changes of blood oxygenation are abrogated by the infusion of bone marrow derived cells**. **A**. Change from baseline perfusate oxygen tension expressed in mmHg (means ± SE) in six experimental groups over the two-hour experimental period. Perfusate blood pO_2 _was measured with a blood gas analyzer. From the infused cell, buffy coat and CD45(+) have the weakest protective effect compared to total BMCs and cultured CD45neg cells. Statistical analysis was done by 2-way ANOVA and Tukey-Kramer for post hoc test. ANOVA, analysis of variance.

Systemic administration of endotoxin also resulted in arterial hypoxemia. Those values are shown over the course of the endotoxin reaction in Figure 4. Arterial hypoxemia following endotoxin was attenuated significantly by administration of either total autologous BMCs, non-hematopoietic CD45(-) cells or swine cultured CD45neg cells. Some average effect was also seen with either buffy coat or CD45 (+) cells, but these were not significantly different from endotoxin alone.

### Infusion of fresh autologous BMC decreased endotoxemia-induced lung edema

**Figure 5:**
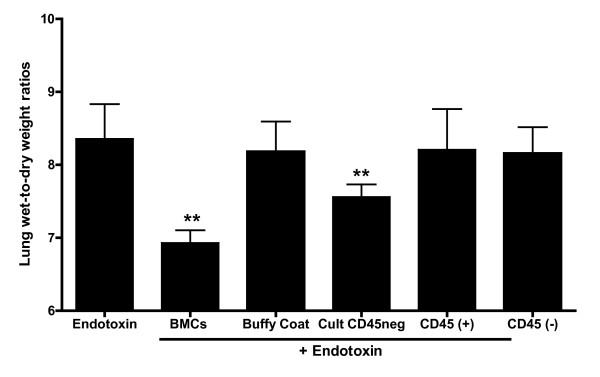
**Infusion of BMC reduces pulmonary edema induced by systemic administration of endotoxin**. As a control lung fibroblasts and buffy coat were added instead of BMC. CD45 (-) cells significantly reduced but did not prevent pulmonary edema induced by endotoxin (*n *= 4 to 5 in each group, *P *<0.05. **). BMC, bone marrow nuclear cells.

As we have reported previously, endotoxin causes a large and significant increase in lung water content [24-30]. Lung wet to dry weight ratios in the experiments reported here are shown in Figure 5. For reference, as has been published by our group, average values in control experiments are approximately 7 [23]. Endotoxin caused pulmonary edema that was significantly attenuated by the infusion of autologous BMCs and swine cultured CD45neg. The lung wet-to-dry weight ratios were not significantly different from the endotoxin group in groups receiving either the subpopulations of CD45(+), CD45(-) or buffy coat.

### Modification of gene expression in the liver by infusion of bone marrow cells

Since previous studies showed that maximal activation of lung pro-inflammatory responses required the presence of the liver, we analyzed gene expression in liver samples after administration of endotoxin. Table 1 lists all of the identified genes that were at least two fold higher in the post endotoxin sample than in samples from animals that did not receive endotoxin. Potential candidate mediators of liver-dependent lung injury are highlighted in the table. Analysis of the data using the Integrity Systems software which links gene expression patterns to biologic pathways, showed the major association with immune/inflammation pathways. Interestingly, of the genes expression of which was increased by endotoxin, 20 were identified as belonging to the canonical acute phase response pathway.

**Table 1:** Hepatic gene expression 30 minutes after endotoxemia normalized to control (no endotoxin).

Encoded protein	Fold Increase after LPS
MHC class II	62
Serine protease inhibitor	19
MHC class I	16
*AMCF-1 *	*14*
*Tumor necrosis factor (TNF) *	*14*
*Prointerleukin-1 beta (IL1B) *	*11*
*Lysozyme (LYZ) Hydroxymethylgluatryl-CoA *	*6*
Synthase (HMGCS2)	5
T cell receptor alpha chain (TCR-a)	5
*Interleukin-6 (IL6)*	*4*
*Interleukin-1alpha (IL1A) *	*4*
*Chemokine ligand 2 (CXCL2) *	*4*
T cell receptor beta (TCR-b)	4
20-beta-hydroxysteroid dehydrogenase (CBR1)	3
C-JUN protein (C-JUN)	3
MHC class II SLA-DRB-c	3
Complement C7 (C7)	3
Heparin binding protein 61	3
Glutathione S-transferase (GST)	3
Prostaglandin G/H synthase-2 (PGHS-2)	3
*RANTES protein (RANTES) *	*3*
*MIP-1 beta protein (MIP-1BETA) *	*3*
Antimicrobial protein hepcidin(HAMP)	3
Interleukin-1 beta converting Enzyme (CASP1)	3
Killer cell lectin-like receptor(KLRK1)	3
CD45 antigen isoform 1 precursor (CD45)	2
*Endothelin (EDN) *	*2*
*Macrophage inflammatory protein 1 alpha (CCL3) *	*2*
Tissue factor	2
TcR gamma chain	2
Plasminogen activator (PLAU)	2
Complement C1qA (C1QA)	2
Heat shock protein 70.2 (HSP70.2)	2
Neuron-derived orphan receptor alpha (NOR-1)	2
Chemokine C-C motifreceptor 5 (CCR5)	2
Complement C1qB (C1QB)	2
SLA-DQ beta1 domanin	2
Beta-2 microglobulin (B2M)	2
TACSTD1	2
CD8 antigen alpha polypeptide (CD8A)	2
Transcription factor PU.1 (PU.1)	2
NF-k gene enhancer inhibitor (NFKBIA)	2

Identified genes with expression increased 2-fold or more; some potential circulating mediators of lung injury are in italics.

**Figure 6:**
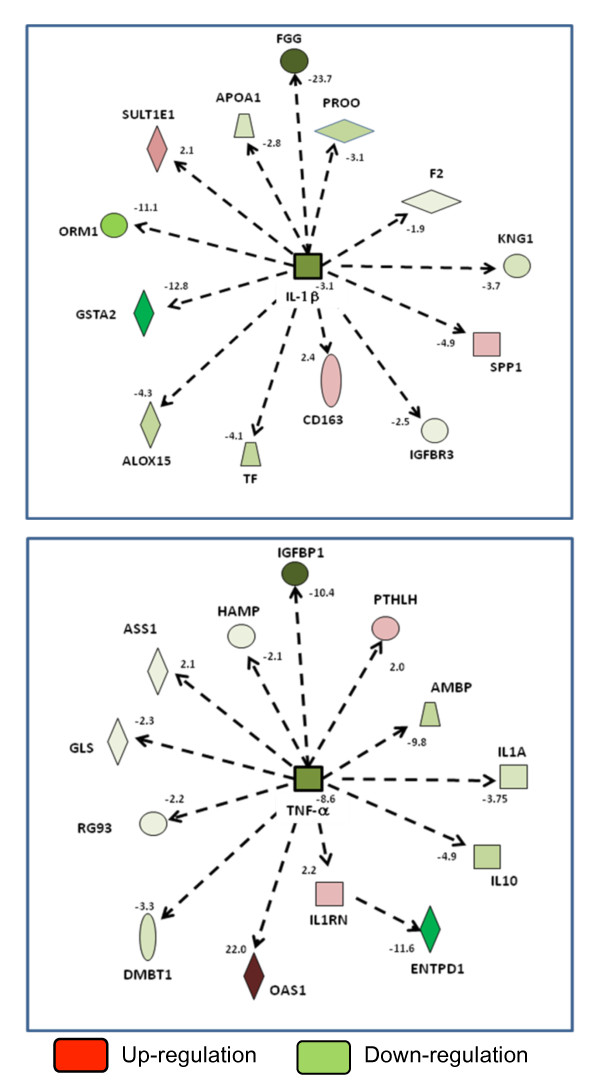
**Immune/inflammation network of genes in the liver whose expression was altered by the infusion of bone marrow derived cells were analyzed using Ingenuity Software**. Response to endotoxin in the presence of bone marrow cells relative to responses to endotoxin alone. Red = marked increase, Pink = increase, Green = decrease. Numbers are fold difference from the endotoxin only control sample.

**Figure 7:**
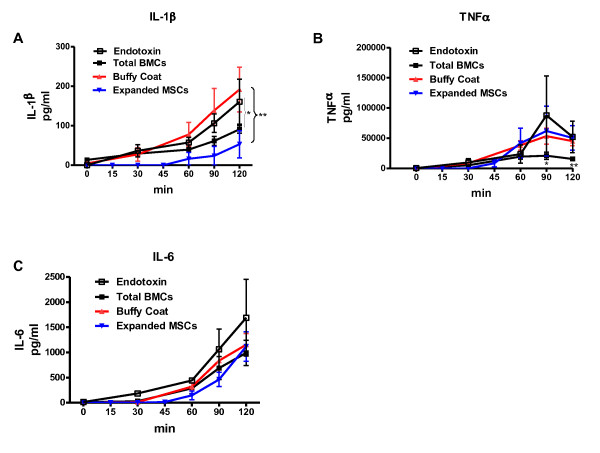
**Administration of freshly isolated whole bone marrow nuclear cells (BMC) altered the systemic inflammatory response to endotoxin**. Compared to responses with buffy coat cells, sixty minutes after infusion of BMC, there was a significant suppression in endotoxin-induced increased systemic concentrations of pro-inflammatory cytokines (**A**) IL-1β and (**B**) TNF-α. There was no attenuation of endotoxin induced release of IL-6 by any of the cells administrated (**C**). (*n *= 6 in each group, **P <0.01, *P <0.05).

Figure 6 shows the effects of BMCs on endotoxin-induced hepatic gene expression, both the fold effect (that is, the ratio of expression with BMCs and endotoxin to expression with endotoxin alone) and the network analysis of gene expression. These results of gene expression implicate IL-1β and TNF-α as prime candidates for the liver-induced lung injury seen after endotoxin. To confirm these results we measured levels of these two cytokines, together with IL-6 as the other inflammatory cytokine involved on ARDS, in the perfusate at baseline and at 15-minute intervals following endotoxin administration and infusion of autologus BMCs, buffy coat and swine cultured CD45neg cells (Figure 7). Endotoxin induced progressive increases in all three cytokines, with particularly high levels of TNF-α. Total BMCs significantly attenuated the TNF-α and IL-1β responses whereas expanded cultured CD45neg cells mainly diminished IL-1β levels. Buffy coat cells had no effect on any of these pro-inflammatory responses. Levels of IL-6 were not affected significantly by the infusion of any type of cell.

## Discussion

One reason for the high morbidity and mortality of ARDS is the lack of effective treatment available. Currently, the only existing therapy is limited to supportive care. Since the introduction of ventilation with low tidal volumes there has been a significant decrease in mortality and an increase in the number of days without ventilator use, which often results in further injury to the lungs [31]. Peak end-expiratory pressure (PEEP) is also an essential component of mechanical ventilation that decreases repetitive opening and closing during the respiratory cycle and opens collapsed alveoli decreasing intrapulmonary shunt. Other ventilatory strategies used in ARDS include recruitment maneuvers, prone position and high frequency ventilation, although the role of these strategies is not well defined and improvement in mortality has not been demonstrated [31].

ARDS can be associated with different clinical disorders directly affecting the lungs (pneumonia or pulmonary contusion) or systemic diseases affecting the lung through the bloodstream (sepsis, severe trauma or acute pancreatitis). Regardless of the cause of ARDS, the alveolar epithelium and capillary endothelium are affected, leading to an increase in permeability that allows fluid to accumulate in the alveolar space (alveolar edema). Loss of epithelial integrity also disrupts alveolar clearance and production of surfactant [32-34]. The extensive alveolar and endothelial damage causes an influx of circulating inflammatory cells that secrete pro-inflammatory cytokines such as TNF-α, IL-1β and IL-6.

A novel *ex-vivo *swine model developed by our group permits the measurement of lung pathophysiology and the simultaneous collection of lung and liver tissue for histologic and molecular comparisons during the early phase of the response to endotoxemia. In this preparation, endotoxemia causes a liver-dependent inflammatory response and severe lung injury and dysfunction [23]. We chose swine as an experimental animal because, like humans, they are especially sensitive to endotoxin and the pathophysiology of the response appears to be similar to that in humans. The model has some limitations as does any acute model, where the physiology can be monitored only for a short period of time before there is a deterioration of the organs.

Here, we find that infusion of freshly isolated suspensions of autologous whole bone marrow nuclear cells, the subpopulation of non-hematopoietic bone marrow cells CD45(-) or *in vitro *expanded adherent swine CD45neg cells, have a moderating effect on the response to endotoxemia. As we expected, the greatest effects were seen with swine cultured CD45neg cells. We and, later, other groups, reported findings in mice that are consistent with the observations that we report here [3,16,35-37]. The ability of the infusion of bone marrow mononuclear cells to prevent organ injury is well known including in the lung [38,39]. In a recent publication, bone marrow derived nuclear cells were used to prevent chronic obstructive pulmonary disease/pulmonary emphysema in rats [40]. A similar rationale has been used to design a clinical trial for emphysema [41]. The present work, to our knowledge, is the first evidence in a clinically relevant lung injury model in a large animal preparation suggesting that freshly isolated autologous bone marrow nuclear cells might have a possible role in therapies for patients with acute lung injury.

The effects we observed as a consequence of cell infusion are on the very early endotoxin response and do not appear to require transdifferentiation into a lung-type cell. These effects were accompanied by an increase in the endotoxin-induced expression of genes encoding anti-inflammatory cytokines and decreased endotoxin-induced expression of genes encoding several pro-inflammatory cytokines in the liver compared to the effects of endotoxin at thirty minutes after endotoxemia. An analysis of the involved cytokine networks indicated that the principal effects of bone marrow cells on the endotoxin molecular response in the liver were on networks in which TNF-α and IL1-β play pivotal roles.

A transient inflammatory response is critical to the body's defense against infectious and other toxic insults. Injury of the lungs and other organs consequent to sepsis in humans and several other animal species appears to result from a dysregulated inflammatory response so that intense inflammation is generalized and persistent [24,42,43]. In cell therapy, several factors appear to contribute to the termination of acute inflammation, including generation of anti-inflammatory cytokines (for example, IL-10) [44-46], cytokine inhibitors [47], antibacterial peptides [48] and growth factors that induce protection of the alveolar epithelial cells [49-51].

In swine, endotoxemia results in increased circulating concentrations of several cytokines and growth factors [23]. Although administration of bone marrow cells did not entirely prevent these responses, they changed the pattern of cytokine responses, significantly decreasing generation of Th1 pro-inflammatory cytokines without decreasing the anti-inflammatory Th2 cytokine IL-6, preventing lung tissue responses to endotoxemia and lung edema. Those responses were temporally coincident with decreased circulating levels of pro-inflammatory cytokines (TNF-α and IL-1β) that are known to affect inflammatory cell trafficking.

## Conclusions

In summary, results presented in the present work suggest that fresh, autologous non-cultured bone marrow nuclear cells, including non-hematopoietic bone marrow derived cells, have the ability to suppress the endotoxin-induced systemic inflammatory response at similar levels to what is observed when *in vitro *expanded heterologous mesenchymal stromal cells are used. Expanded or freshly isolated autologous bone marrow mononuclear cells could prove to be a novel approach to therapy for some acute and chronic lung diseases.

## Abbreviations

ARDS, Acute Respiratory Distress Syndrome; BMC, whole bone marrow cell suspensions; BSA, bovine serum albumin; Cultured CD45neg cells, *in vitro *expanded swine CD45 negative adherent allogeneic bone marrow cells; EDTA, ethylenediaminetetraacetic acid; ELISA, enzyme-linked immunosorbent assay; FACS, fluorescence-activated cell sorting; FBS, fetal bovine serum; FITC, fluorescein isothiocyanate; IL, interleukin; LPS, lipopolysaccharide; MPO, myeloperoxidase; PBS, phosphate buffered saline; TNF-α, tumor necrosis factor alpha.

## Competing interests

The authors declare that they have no competing interests.

## Authors' contributions

MR and KLB conceived, interpreted and analyzed the data and drafted the manuscript. RP and NT were responsible for swine experiments, collection and assembly of data. CC and SI executed the ELISA and FACS assays and they did the expansion of swine adherent CD45neg and consequent data analysis and interpretation. AS and AM made substantial contributions in the conception and design of the experiments and critically revised the content of the completely revised manuscript. LM, MB and NC were responsible for tissue processing, staining and analysis. All authors read and approved the final manuscript.
